# Woman's Work in Medicine after the War

**Published:** 1917-09-08

**Authors:** 


### WOMAN'S WORK IN MEDICINE AFTER THE WAR.

### Memory Pictures and the Worker.

IN our Educational Number last year we felt it needful to comment upon some remarks which appeared in the *Evening Standard and St. James's Gazette* regarding woman's place in the medical world. This year there is no need for many of the contentions which we then advanced. We had long recognised that in the nursing world

> Among the many rare and special gifts
> That in the female sex are found to sit
> This one is chief.—WEEVER.

But it was a little trodden path in the domain of medicine which was to mark her fitness to

> Give best advice and show most ready wit.

"It had hitherto been the province of man to chew and think and sift," and even to him, with centuries of training, the road was none too clearly marked. We recognised that in the disasters of war much progress would be made by man along the ways mapped out by military needs, but at some sacrifice to the art of medicine and the daily requirements of the civil population. We acknowledged with grateful thanks the skill of the woman workers in the laboratory, searching for the causes of disease, and in the preparation of protective or curative serums. But we also ventured to forecast that after the war much of this work would furnish the living wage for such men as had been crippled in the firing line, and were unfitted for other posts. Only those women who had made themselves indispensable by special work could be retained—the average worker must learn that, having "done her bit," "A woman's noblest station is retreat" (Lyttleton). But any such retreat must be strategic—it can mean no more than a reassembling of forces pending a new distribution.

We still think, as we did a year ago, that the realm of surgery will be chiefly tenanted by man, but not to the exclusion of woman's work in special branches. In surgery observation and action follow one another quickly, and that rapid succession is more akin to the life of those who have spent years on war service than to the conditions of home or private work.

Man in his war work and woman in her new work are quite truly writing history; but at the same time they are forming their memory for future use. In his recently published reminiscences Tagore contends that the two correspond, but that they are not one. "Over Life's outward aspect passes the series of events and within is being painted a set of pictures." To men serving at the Front this experience means meeting surgical conditions produced by calculable causes and with suitable provision. To woman, by needs relegated to the second line, it means the carrying on of what has been begun with the adjunct of improved environment. These factors form the memory pictures of the worker and fit him for its continuance. The experience which is the basis of the art of medicine is more personal and more widely spread. It leads up to the recognition of disease which is more hidden in its origin and less obvious in its causation, and it takes more account of the personality of the patient. It has been the work of centuries and is the inheritance of generations; but there are still some who regard it with favour, even if there be few who would not speak of it in Tennyson's words quoted by Cecil Rhodes at the end of his strenuous life—"So much to do! So little done!" It has been the safeguard of the relations between doctor and patient, and even if it be no more than an art, its result has favoured security and comfort.

The beginnings of disease have been tracked and checked by means less scientific than prevail at present in the war areas, and if some such organisation as was suggested by Dr. Hawthorne to the Metropolitan Branch of the British Medical Association can be realised, there is no reason why the distinction between the sexes pictured in the lines quoted from the writings of three centuries ago should not vanish; why rivalry should not give place to comradeship and good will, and make opportunity, cultivation, and development the natural sequence of events. The record of service detailed by Dr. Mary H. Frances Ivens in a recent number of the *British Medical Journal* is the story of the indispensables, for whom there must be no retreat. She and her like have been revelations of the height to which women can rise under unprecedented conditions; but with the cessation of the war the work of the majority will lie in the less brilliant and perhaps more arduous conditions of private practice. The younger men, who have not yet formed their full gallery of memory pictures in civil practice, and who have spent their most active years on war service, will, to use Tagore's expression, have "put in the background that which was to the fore," and it will remain for woman "to bring to the front that which was behind." The care of the crippled and the otherwise injured after the war will be an urgent but gradually diminishing quantity. The lesser ailments which formerly prevailed and frequently laid the foundations of grave disease will increase in amount. Public health measures will reach them with a firmer grip than of yore, but their range must be lengthened beyond the location which exists with troops in the field, and that will require many women helpers.

The past year has made it obvious that medical work has no injurious influence upon a woman's health. We hear of medical women who have spent their sessions in study, taking up agricultural work as a holiday pastime. We have seen unemployed women fall victims to the nervous, cardiac, and thyroid troubles in far greater proportion than those who have spent their energy in the study of health and the ministry of disease. Even business, bank work, and arduous domestic duties have failed in the fuller award. We look forward during the coming year to the wider recognition of woman's helpful work in the medical world.

